# A Comparative Analysis of Polyfunctional T Cells and Secreted Cytokines Induced by Bacille Calmette-Guérin Immunisation in Children and Adults

**DOI:** 10.1371/journal.pone.0037535

**Published:** 2012-07-19

**Authors:** Nicole Ritz, Madeleine Strach, Carmen Yau, Binita Dutta, Marc Tebruegge, Tom G. Connell, Willem A. Hanekom, Warwick J. Britton, Roy Robins-Browne, Nigel Curtis

**Affiliations:** 1 Department of Paediatrics, The University of Melbourne, Parkville, Australia; 2 Murdoch Children’s Research Institute, Parkville, Australia; 3 Infectious Diseases Unit, Department of General Medicine, The Royal Children’s Hospital Melbourne, Parkville, Australia; 4 South African Tuberculosis Vaccine Initiative, Institute of Infectious Disease and Molecular Medicine and School of Child and Adolescent Health, University of Cape Town, Cape Town, South Africa; 5 Centenary Institute of Cancer Medicine and Cell Biology, University of Sydney, Camperdown Australia; 6 Department of Microbiology and Immunology, The University of Melbourne, Parkville, Australia; 7 Department of Medicine, University of Sydney, Camperdown Australia; National Institute of Infectious Diseases, Japan

## Abstract

BCG vaccine is one of the most commonly-administered vaccines worldwide. Studies suggest the protective efficacy of BCG against TB is better for children than for adults. One potential explanation is that BCG induces a better protective immune response in children. Twenty six children and adults were immunised with BCG. The proportion of Th1-cytokine-producing mycobacterial-specific T cells, and the concentrations of secreted cytokines, were measured before and 10 weeks after BCG immunisation. A significant increase in the proportion of mycobacterial-specific cytokine-producing T cells was observed in both age groups. After BCG immunisation, children and adults had comparable proportions of mycobacterial-specific polyfunctional CD4 T cells when measured relative to the total number of CD4 T cells. However, relative to the subset of Th-1-cytokine-producing CD4 T cells, the proportion of polyfunctional cells was greater in children. Concentrations of secreted cytokines were comparable in children and adults. These findings suggest that the mycobacterial-specific cell-mediated immune response induced by BCG immunisation in children and adults is similar. The implication of a shift to a more polyfunctional immune response within the Th1-cytokine-producing CD4 T cells in children is uncertain as this aspect of the immune response has not been assessed as a potential correlate of protection against TB.

## Introduction

Bacille-Calmette-Guérin (BCG) is one of the most commonly administered vaccines worldwide with more than 100 million doses given each year. Studies investigating the protective efficacy of BCG against tuberculosis (TB) suggest protective efficacy is better for children than for adults [1], [2]. One potential explanation is that BCG induces a better protective immune response in children. Although there is evidence that age at immunisation influences the immune response to BCG, the two most recent studies addressing this issue compared the immune response in different age groups both in the first year of life [3], [4]. Only one previous study, from the Gambia, has compared the immune response in BCG-immunised children and adults [5]. However, in this study the adults had been BCG immunised many years earlier whereas the children had been immunised only two months prior to assessment. Consequently, in the adult group, waning immunity or intervening exposure to *Mycobacterium tuberculosis* may have influenced the results. Therefore, currently available data do not answer the question whether there is a difference in the immune response between BCG-immunised adults and children when measured at a similar time interval after immunisation in both age groups.

A number of studies in either children or adults have investigated the complex immune response induced by BCG-immunisation. In children, this has most commonly been studied in infants. Neonatal BCG immunisation has been shown to induce antigen-specific interferon (IFN)-γ-producing CD4 and CD8 T cells [6], [7], [8], [9], [10], proliferation of CD4 [11], [12], CD8 [7] and γ/δ T cells [10], [11], cytotoxicity in CD8 T cells [7], [12], secretion of a variety of cytokines and chemokines [8], [9], [11], [12], [13], [14], [15], and more recently, the induction of mycobacterial-specific polyfunctional CD4 T cells [10], [16]. Polyfunctional CD4 T cells simultaneously producing IFN-γ, interleukin (IL)-2 and tumor necrosis factor (TNF)-α, or a combination thereof, have recently attracted attention as potential correlates of protection against TB [17].

Few studies have investigated the immune response to BCG in recently-immunised adults. In this age group, BCG has been shown to induce a mycobacterial-specific IFN-γ response [18], [19], [20], [21], [22], T cell proliferation [18], [19], [20], [21], [22] and cytotoxicity of CD8 T cells [21], [23].

Differences in the immune response induced by BCG between children and adults might explain the difference in protective immunity conferred by BCG in these age groups.

## Materials and Methods

### Ethics Approval

Approval for this study was obtained from the respective human research ethics committees of the Royal Children’s Hospital and The University of Melbourne, Australia. Written informed consent was obtained from parents or participants.

**Figure 1:**
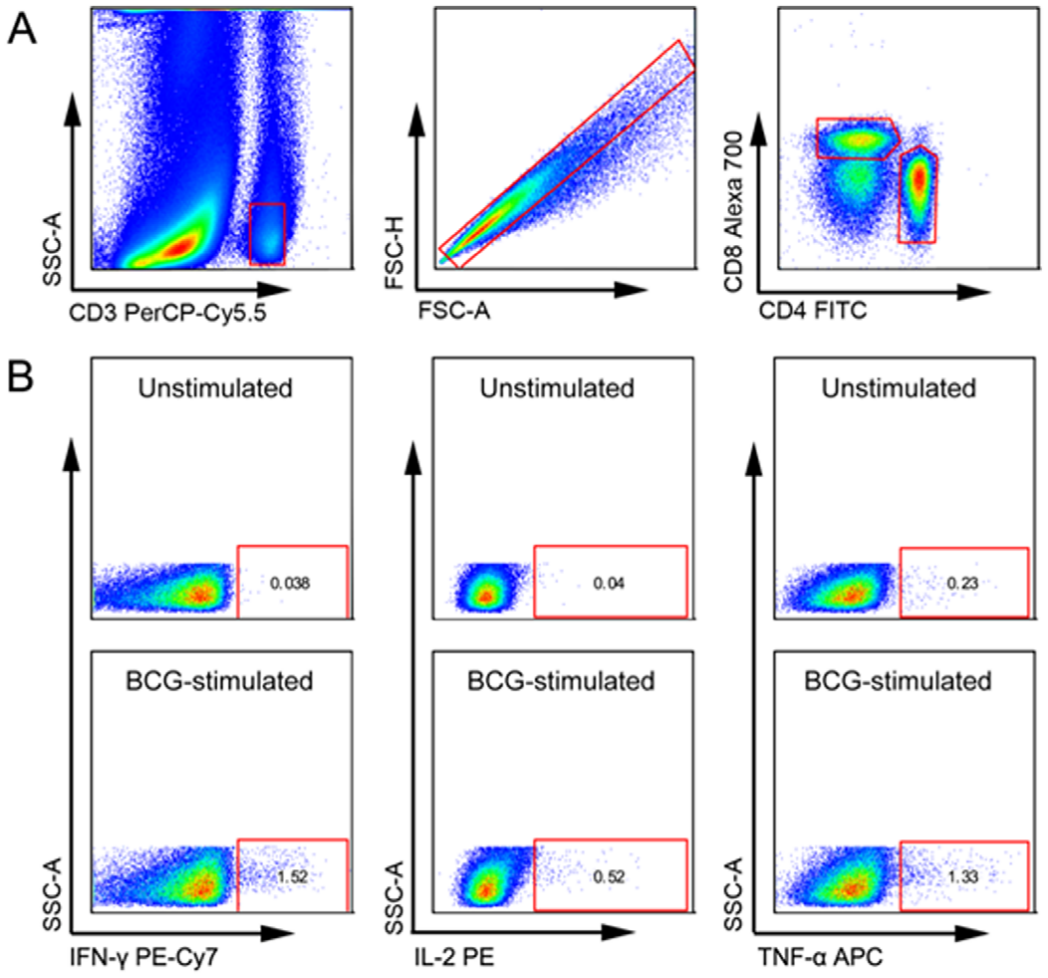
Gating strategy. (A) Selection of populations containing single cell CD4 or CD8 T cells. Each subsequent panel shows only the population of interest that has been selected from the gate on the previous plot. (B) Selection of CD4 T cells producing the cytokines of interest shown in an unstimulated and a BCG stimulated sample from one individual. The same gating strategy was used for CD8 T cells (not shown). Boolean gating was used to calculate proportions of polyfunctional T cells.

### Study Participants and Blood Collection

Healthy children under two years of age, who were to be given BCG immunisation for intended travel to countries with high TB incidence, were recruited at the Royal Children’s Hospital Melbourne. Adult volunteers were recruited from medical students at the University of Melbourne. After obtaining written informed consent, intradermal BCG vaccine (BCG-Connaught, Sanofi Pasteur, Toronto, Canada) was administered in the left deltoid region by a using a dose of 0.05 ml in children under 12 months of age and 0.1 ml after the first year of life. A tuberculin skin test (TST, 5 TU Tubersol, Sanofi Pasteur, Toronto, Canada) was done before immunisation in individuals over six months of age to exclude previous *M. tuberculosis* infection. In the adult volunteers, an IFN-γ release assay (QuantiFERON-TB Gold In-Tube, Celestis, Carnegie, Australia) was also done. Blood was collected before, and 10 weeks after BCG immunisation.

### Antigens and Antibodies for *in vitro* Assays

Lyophilised BCG was reconstituted to a concentration of 100×10^6^ CFU/ml by adding sterile water and Roswell Park Memorial Institute media (Invitrogen, Mulgrave, Victoria, Australia) and used at a final concentration of 1.6×10^6^ CFU/ml. Purified protein derivative (PPD, Batch RT50, Statens Serum Institute, Copenhagen, Denmark) was used at a concentration of 20 µg/ml. Staphylococcal enterotoxin B (SEB, Sigma-Aldrich, St. Louis, MO, USA) was used at a concentration of 10 µg/ml as a positive control. Co-stimulatory antibodies anti-CD28 and anti-CD49d (both from BD Biosciences, San Jose, CA, USA) were used each at a concentration of 1 µg/ml [24].

### Whole Blood Intracellular Cytokine Assay

One ml of heparinised blood was incubated within one hour of collection with BCG, PPD, SEB or media alone in the presence of co-stimulatory antibodies CD28 and CD49d at 37°C for 12 hours. After 7 hours, 200 µl of plasma were removed and cryopreserved, and Brefeldin A (BfA) (Sigma-Aldrich) was added at a concentration of 10 µg/ml. After 12 hours samples were cooled to room temperature and harvested within 10 hours using ethylenediaminetetraacetic acid (EDTA, Sigma-Aldrich) at 2 mM. Cells were incubated with FACS lysing solution (BD Biosciences) before cryopreservation at −80°C.

### Cell Staining and Flow Cytometric Analysis

Cryopreserved cells were thawed and permeabilised with Perm2 Solution (BD Biosciences) for 10 minutes. Cells were then washed with PBS staining buffer containing 0.5% bovine serum albumin and 0.1% sodium azide before incubation with fluorochrome-conjugated antibodies for 30 minutes in the dark. The following conjugated antibodies (all from BD Biosciences) were used: anti-CD3 PerCP-Cy5.5 (SK7), anti-CD4 FITC (RPA-T4), anti-CD8 Alexa-700 (RPA-T8), anti-IFN-γ PE-Cy7 (4S.B3), anti- IL-2 PE (MQ1-17H12) and anti-TNF APC (Mab11). The choice and concentrations of fluorochrome-conjugated antibodies had been previously optimised. Flow cytometric acquisition was done on a LSRII flow cytometer (BD Biosciences) with voltages of photomultiplier tube voltages and settings optimised using cytometer setup and tracking beads (BD Biosciences). Automated compensation was calculated with FACSDiva software (version 6.1, BD Biosciences) using anti-mouse and anti-rat Ig kappa compensation beads. For each sample, a minimum of 100 000 CD3 cells were acquired. Flow cytometric analysis was done using Flowjo (version 8.8.6, TreeStar Inc, Ashland, OR, USA). A hierarchical gating strategy was used to select single cell CD4 and CD8 T cell populations (Figure 1 panel A). Gates for cytokine production from blood stimulated with BCG or PPD were set using the unstimulated control. A Boolean combination of gates was used to calculate double- and triple-positive populations. The mean fluorescence intensity was calculated using Flowjo software for all subpopulations. For single- and double-cytokine-producing populations, MFI was analysed only when more than nine cells per population were detected; for triple cytokine-producing cells, MFI was analysed for populations with more than four detected cells.

**Figure 2:**
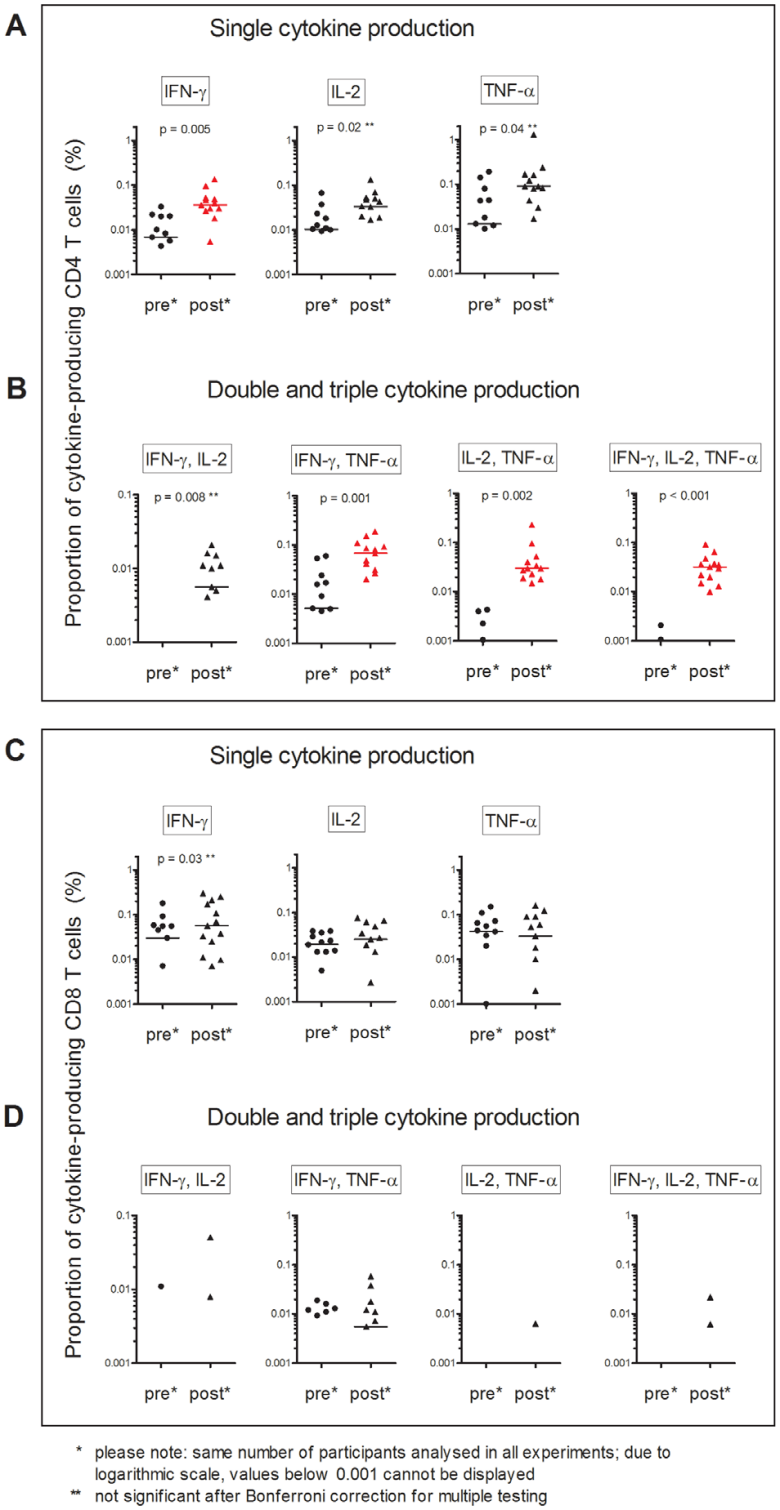
Proportion of cytokine-producing T cells in children before (pre, n = 13) and 10 weeks after (post, n = 13) BCG immunisation. Whole blood was incubated for 12 hours with the antigen BCG to measure proportions of IFN-γ, IL-2 and TNF-α producing CD4 T cells (A) and CD8 T cells (C). Boolean analysis was used to calculate proportions of multifunctional cells producing two or all three Th1 cytokines simultaneously (B, D). Bars indicate medians. Statistical differences with p-values <0.05 are shown. Significant changes after Bonferroni correction are shown in red.

**Figure 3:**
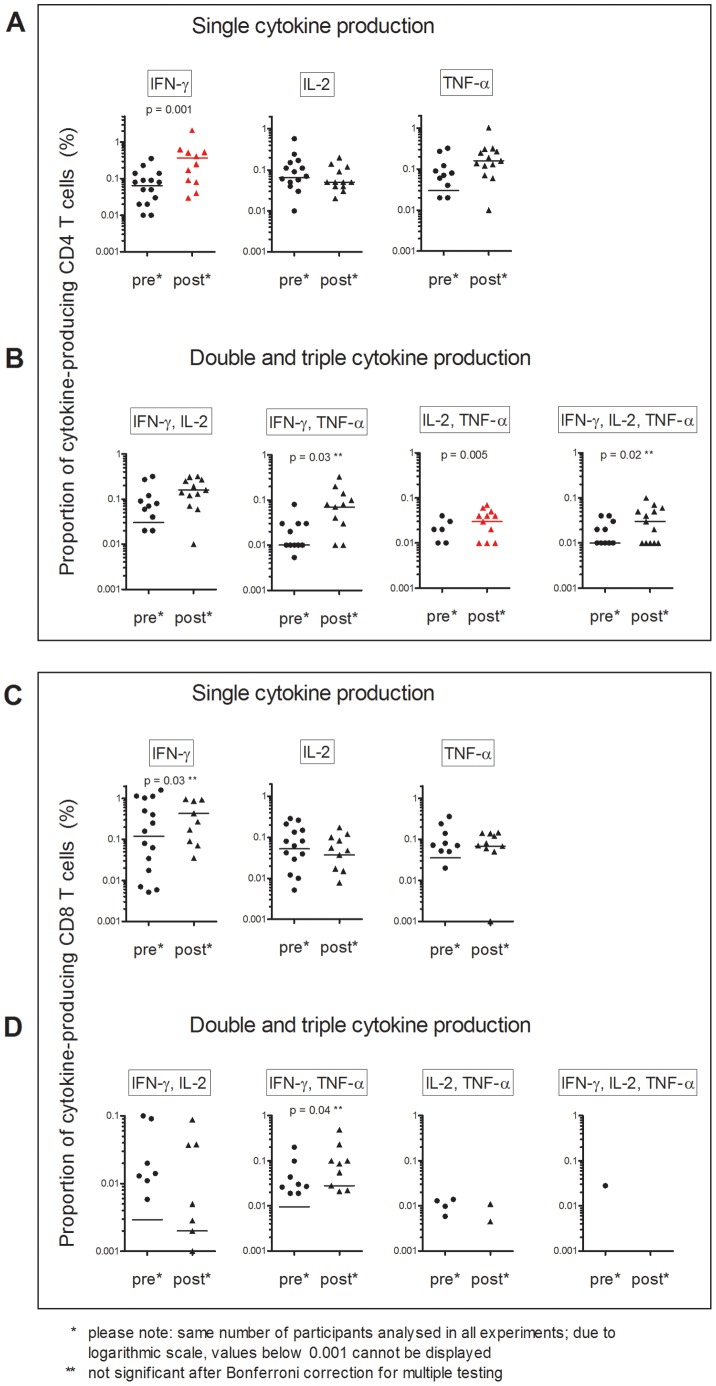
Proportion of cytokine-producing T cells in adults before (pre, n = 16) and 10 weeks after (post, n = 13) BCG immunisation. Panels A to D as for **Figure 2**.

### Cytokine/chemokine Analysis

Cryopreserved supernatants were thawed and analysed using xMAP technology (Milliplex Human Cytokine Immunoassay, Millipore Corp., Billerica, MA, USA) for the following cytokines and chemokines: eotaxin, IL-2, IL-6, IL-10, IL-12 (p40), IL-13, IFN-γ, monocyte chemotactic protein (MCP)-1, macrophage inflammatory protein (MIP)-1β and TNF-α. Pilot experiments showed that IL-4, IL-5 and IL-17 were not produced in detectable concentrations in this assay (data not shown). The manufacturer’s instructions were followed. The provided standards were used to generate standard curves. Samples were added undiluted at a volume of 25 µl for all cytokines and additionally for IL-6, MCP-1, MIP-1β and TNF-α in 1∶20 dilutions. Mixed beads were added and plates incubated on a plate shaker at room temperature in the dark for one hour. After washing plates detection antibodies were added and incubated for 30 minutes followed by another 30 minutes incubation with streptavidin-phycoerythrin solution. The plate was washed, sheath fluid added analysed on a calibrated Luminex 200 instrument (Luminex Corp., Austin, TX, USA) using xPONENT software (version 3.1, Luminex Corp., Austin, TX, USA). For analysis cytokine/chemokine concentrations were background-corrected by subtracting the concentrations detected in the unstimulated sample.

**Figure 4:**
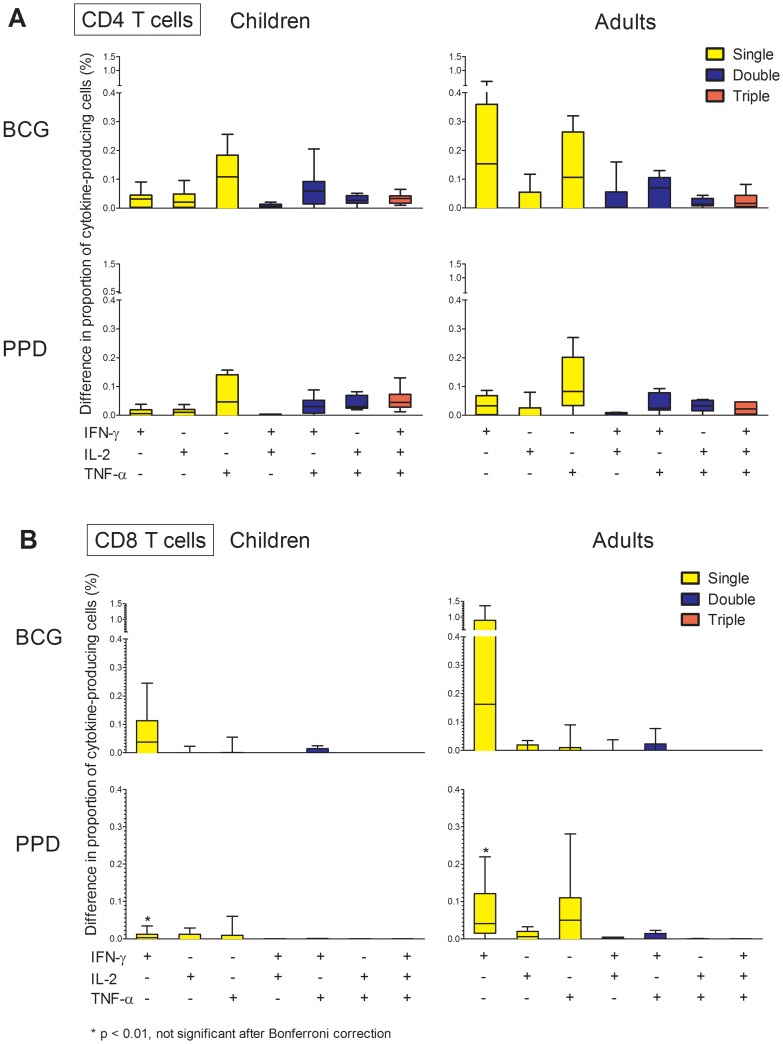
Box plots (with lower quartile, median and upper quartile, whiskers 1.5 IQR) showing median change in the proportion of cytokine-producing subpopulations of CD4 T cells 10 weeks after BCG immunisation in children (n = 13) and adults (n = 13). The proportion of Th1-cytokine-producing subpopulations in CD4 and CD8 T cells was measured following *in vitro* stimulation with the antigens BCG and PPD, in whole blood taken before and after BCG immunisation. The median change for (A) CD4 and (B) CD8 T cells was calculated by subtracting results before BCG immunisation from results after BCG immunisation. Differences were compared using a Mann-Whitney test.

**Figure 5:**
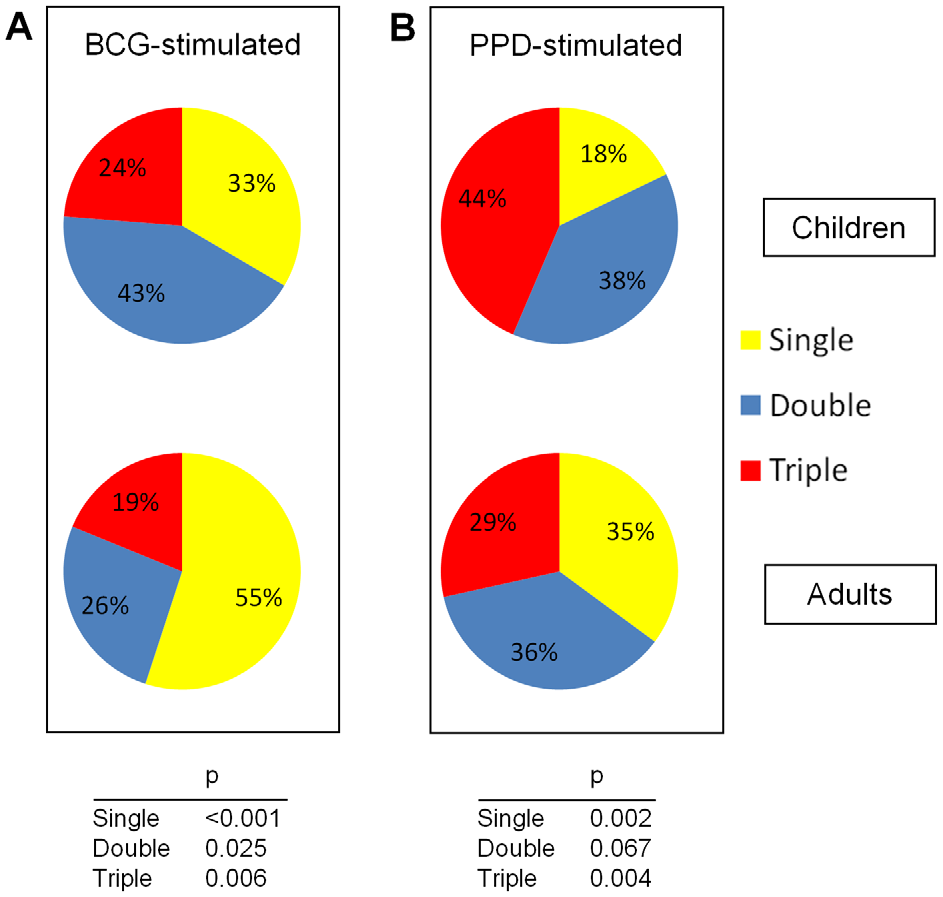
The fraction of single-, double-, and triple- cytokine-producing CD4 T cells relative to the total population of Th1-cytokine producing cells in children (n = 13) and adults (n = 13) following *in vitro* stimulation with antigens (A) BCG and (B) PPD. Differences were compared using a Mann-Whitney test.

### Statistics

For comparison of results before and after BCG immunisation a Wilcoxon matched pairs test was used. For comparison of results in children and adults, a Mann-Whitney test was used. For overall comparisons of MFI a Kruskal-Wallis test was used, and for comparisons of two populations a Mann-Whitney test was used. Significance for all correlations were calculated using a Spearman’s rank correlations coefficient. Statistics were calculated and graphs produced using Prism 5 (Graph Pad Software Inc., La Jolla, CA, USA). A p-value less than 0.05 was considered significant in the initial analysis, and Bonferroni corrections were used to account for multiple testing.

**Figure 6:**
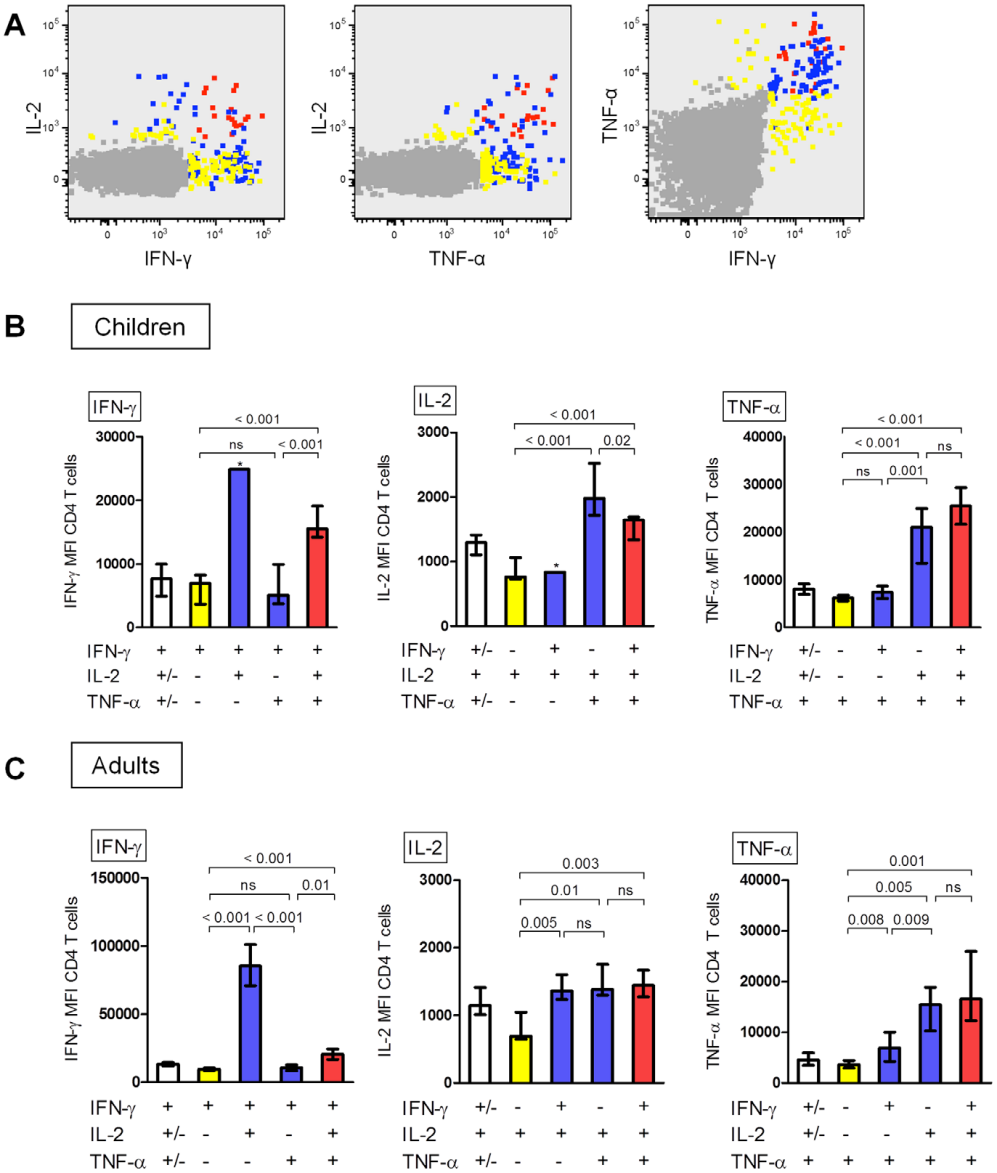
Fluorescence intensity of CD4 T cells following *in vitro* stimulation with the antigen BCG. (A) Fluorescence intensity of single-(yellow), double-(blue), or triple-(red) cytokine-producing CD4 T cells in a representative child 10 weeks after BCG immunisation. Events are plotted on a logarithmic scale. Cells in the top right hand corner are those producing the greatest amount of both cytokines. (B, C) Median fluorescence intensity (MFI) for IFN-γ-, IL-2- and TNF-α-producing CD4 T cell subpopulations following *in vitro* stimulation with the antigen BCG for (B) children (n = 13) and (C) adults (n = 13) 10 weeks after BCG immunisation. White bars show total cytokine-producing populations. Whiskers indicate IQR. Bars with * are based on one value only. Overall comparison was done using a Kruskal-Wallis test and comparisons of two populations were done using a Mann-Whitney test. The numbers above the horizontal bars represent the p-values for comparisons. ns  =  not significant (p>0.05).

**Figure 7:**
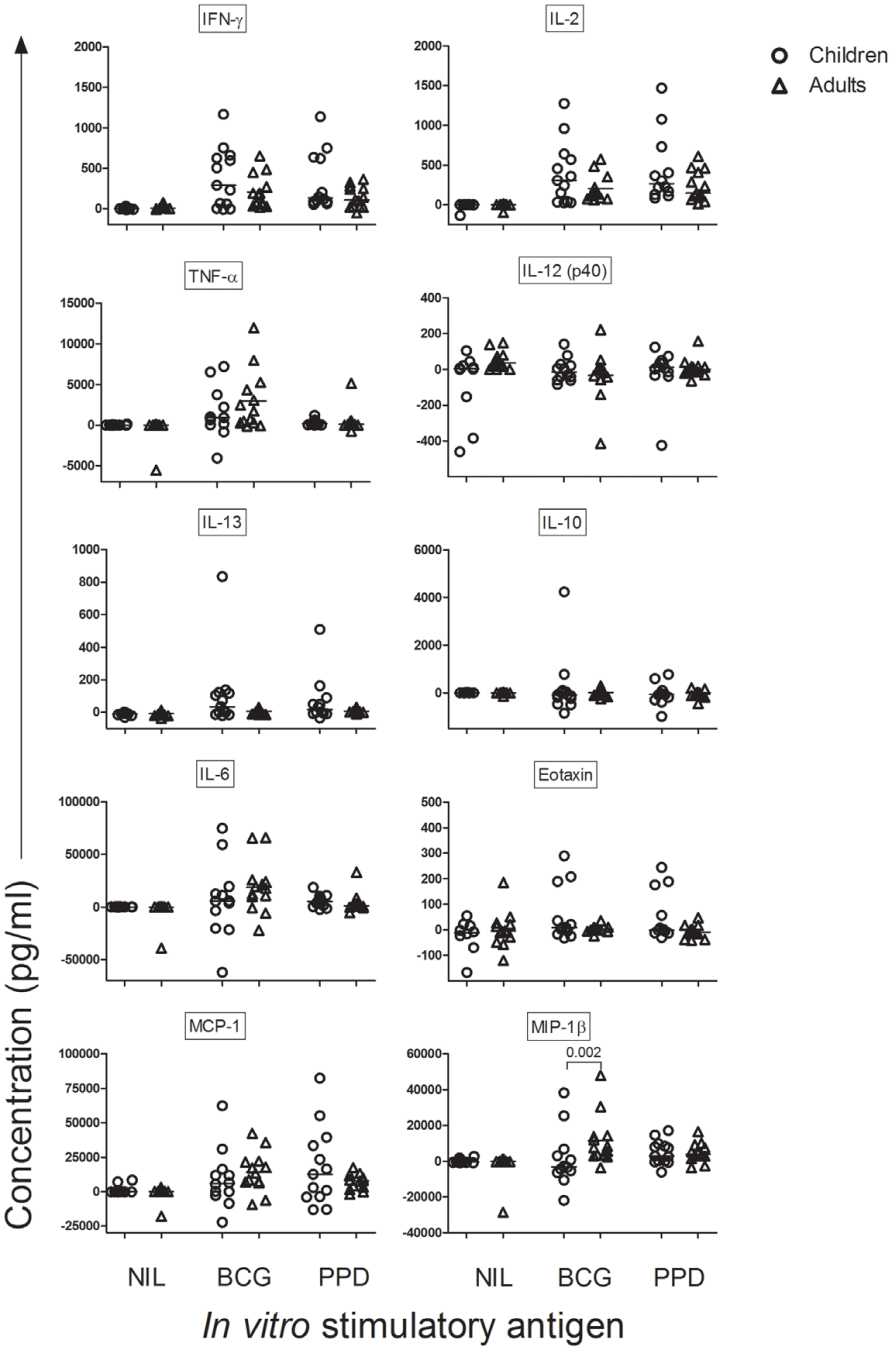
Comparison of the changes in cytokines/chemokines between 13 children (circles) and 13 adults (triangles). Concentrations were determined using xMAP technology following *in vitro* stimulation with the antigens BCG and PPD in samples taken before and 10 weeks after BCG immunisation. Statistical differences were analysed using a using a Mann-Whitney test and p-values <0.05 are shown. Bars indicate medians.

## Results

### Study Participants

Of the 23 children recruited, 13 returned for a second blood sample after BCG immunisation. Their median age at BCG immunisation was 9.6 months (range 6 weeks to 2 years) and four participants were female. Results were negative (<5 mm) for all TSTs performed. Of the 16 medical students recruited, 13 returned for the second blood sample after BCG immunisation. Their median age at BCG immunisation was 24.0 years (range 20.7 to 32.4 years) and nine participants were female. Results were negative for all TSTs (<5 mm) and QuantiFERON-TB Gold In-Tube assays (TB antigen response minus nil control response ≤0.35 IU/ml).

### Proportions of Cytokine-producing T cells before and after BCG Immunisation

In children, the proportions of the seven subpopulations of BCG-specific CD4 T cells producing IFN-γ, IL-2 or TNF-α alone or simultaneously (ie polyfunctional CD4 T cells) was increased 10 weeks after BCG immunisation (Figure 1 panel B, Figure 2 panel A and B). In adults, a similar pattern was seen (Figure 3, panel A and B). Notably, adults had higher proportions of BCG-specific CD4 T cells before BCG immunisation compared to children. In both children and adults, the proportion of Th-1 cytokine-producing BCG-specific CD8 T cells, did not significantly differ 10 weeks after BCG immunisation (Figures 2 and 3, panel C and D).

Similar results were seen in PPD-specific CD4 and CD8 T cells in both children and adults (data not shown).

### Changes in Cytokine-producing T cells in Children Compared to Adults

The median change in cytokine-producing T cell populations was calculated for each participant by subtracting the proportions before BCG immunisation from proportions after BCG immunisation. There was no significant difference between children and adults in the change in proportions of BCG- and PPD-specific CD4 T cells (Figure 4, panel A). For CD8 T cells, there was a greater increase in the proportion of single IFN-γ-producing T cells in adults than in children (Figure 4, panel B). However, this difference was not statistically significantly.

Next we compared the quality of the immune response in children and adults by calculating the proportion of single-, double- or triple-cytokine producing cells within the Th1 cytokine-producing CD4 T cell population (in contrast to within the total CD4 T cell population). Following *in vitro* stimulation with BCG, the proportion of polyfunctional Th1-cytokine-producing T cells was significantly higher in children than in adults (Figure 5 panel A). Following *in vitro* stimulation with PPD, similar results were found (Figure 5 panel B).

### Cytokine-producing Capacity of T cells

Polyfunctional CD4 T cells showed higher cytokine-producing capacity in both children and adults. For example, following *in vitro* stimulation with BCG, cells producing IFN-γ without simultaneous production of IL-2 or TNF-α showed lower mean fluorescence intensity (MFI) for IFN-γ than cells producing two or three of these cytokines simultaneously (Figure 6, panel A). The same was true for IL-2 and TNF-α.

In children, the median MFI was generally greater in double- and triple-cytokine-producing compared to single-cytokine-producing CD4 T cells for IFN-γ, IL-2 and TNF-α following *in vitro* stimulation with BCG (Figure 6, panel B). Similarly in adults, the MFI of triple-cytokine-producing CD4 T cells was significantly greater than the MFI of single-cytokine producing T cells following *in vitro* stimulation with BCG (Figure 6, panel C).

### Cytokines and Chemokines in Supernatants

Next we analysed secreted cytokines and chemokines in supernatants. In accordance with the results from flow cytometry, there was a significant increase in the concentration of IFN-γ, IL-2 and TNF-α after BCG immunisation in both children and adults (Figures S1 and S2). Comparing the median change (concentration before BCG immunisation subtracted from concentration after BCG immunisation) there was no significant difference in the concentration of Th1 cytokines between children and adults (Figure 7).

For all other analysed cytokines and chemokines, there were also no statistically significant differences with the exception of MIP-1β. This chemokine was significantly more increased in BCG-immunised adults than in BCG-immunised children following *in vitro* stimulation with BCG (Figure 7).

Interestingly, in both age groups, supernatants from samples taken before BCG immunisation which were stimulated *in vitro* with BCG had increased concentrations of a number of cytokines (TNF-α, IL-10; IL-6, MCP-1 and MIP-1β) compared to unstimulated samples (Figures S1 and S2).

## Discussion

Investigating differences in the immune response between children and adults is important to understand the immunological mechanisms underlying protective efficacy against TB induced by BCG immunisation. In particular, this may provide an explanation for the difference in protective efficacy afforded by BCG immunisation in children and adults. Our study is the first to compare the BCG-induced immune response in children and adults at the same time interval after immunisation. It is also the first to analyse the BCG-induced immune response in both age groups using a comprehensive panel of immunological assays.

The median change in proportions of the seven subpopulations of Th1 cytokine-producing T cells induced by BCG immunisation was not different between children and adults. However, in children a larger number of polyfunctional T cell subsets showed statistically significant increases in proportions, and these increases were generally greater. This might be partially explained by the higher proportions of Th1 cytokine-producing T cells in adults before immunisation.

A similar spectrum of T cell subpopulations induced by BCG immunisation was recently reported in a study in infants immunised at birth in South Africa [16]. The results from this study are consistent with our finding but the proportions of TNF-α-single and IFN-γ/TNF-α-double cytokine producing cells were lower in the South African study. This may be attributed to the lower age of the children in the South African study as the frequency of TNF-α-producing T cells has been shown to increase with age [25].

A number of recent studies have investigated polyfunctional CD4 T cells as potential correlates of protection against TB with conflicting results. Some studies in TB and human immunodeficiency virus co-infected individuals show an association between mycobacterial-specific polyfunctional T cells and protection against TB [26], [27], [28], [29]. In contrast, other studies in humans suggest that mycobacterial-specific polyfunctional T cells are associated with active TB disease [30], [31]. Also, a case control study in BCG-immunised infants in South Africa failed to correlate polyfunctional T cells with protection [10]. Studies investigating novel TB vaccines in mice, however, have found a correlation between mycobacterial-specific polyfunctional T cells (in lung tissue, peripheral blood mononuclear cells or spleen) and protection [32], [33], [34], [35].

The suggested mechanism underlying the concept that polyfunctional CD4 T cells are key to protection against intracellular pathogens, including *M. tuberculosis*, is that the simultaneous production of IFN-γ and TNF-α is synergistic in killing intracellular pathogens [36] and this effect is further enhanced by IL-2-induced proliferation of these cells. In addition, polyfunctional CD4 T cells produce greater quantities of each Th1 cytokine compared to single-cytokine-producing cells. Polyfunctional CD4 T cells have been shown to produce more IFN-γ, TNF-α and IL-2 in adults [37], [38], [39] but, to our knowledge, this is the first study in children showing that polyfunctional T cells have higher MFIs and therefore higher cytokine-producing capacity for all three Th1 cytokines. MFI data must be interpreted cautiously as they are sometimes based on few cells. However, in our study, MFI results were only analysed when there were sufficiently high numbers of CD3 cells.

Relative to the subset of Th1-cytokine-producing CD4 T cells, the proportion of polyfunctional T cells was greater in children than in adults. In the studies showing that polyfunctional T cells are correlates of protection [33], [37], [40], polyfunctional T cells have been expressed as a proportion relative to the total number of CD4 T cells. Therefore, it remains unknown whether the shift to a more polyfunctional immune response within the Th1-cytokine-producing cell population observed in children is associated with better protection. Evaluation of the memory phenotype of T cells within the Th1-cytokine-producing cell population in future studies would help discern whether the difference in the polyfunctional T cell response between children and adults is attributable to age-related changes in proportions of naïve and memory phenotypes.

In our study there was no difference between the median concentrations of Th1-cytokines in supernatants in children and adults after BCG immunisation. This finding is consistent with the absence of a difference in the change in proportions of cytokine-producing, and in particular polyfunctional CD4 T cells, measured by flow cytometry. This finding is also in keeping with a previous study, which did not find a difference in the frequency of IFN-γ-producing T cells and the concentrations of IFN-γ, IL-4 and IL-5 in supernatants between children and adults [5]. However, in this study the immune response in adults was measured several years after BCG immunisation and other factors including waning of immunity are likely to have influenced the findings.

The implication of the observed lower MIP-1β induced by BCG immunisation in children compared to adults is unclear. The role of MIP-1β in protection against TB is believed to be the recruitment of monocytes and lymphocytes to the site of infection and thereby accelerating granuloma formation [41]. MIP-1β has also been shown to have antimycobacterial properties in macrophages [42]. One potential reason for our finding is decreased MIP-1β production attributable to immaturity of monocytes and dendritic cells in children.

A number of important *in vitro* effects of BCG were also observed in our study. Concentrations of TNF-α, IL-6, IL-10, MCP-1 and MIP-1β were increased following *in vitro* stimulation with BCG in samples from children and adults taken **before** BCG immunisation. This finding could be attributed to previous exposure of the study participants to tuberculous or non-tuberculous mycobacteria. However, exposure to mycobacteria in Australia is limited, and moreover all participants over the age of six months had a negative TST and/or IFN-γ release assay result. It is therefore more likely that the increased concentrations of these cytokines and chemokines are the result of an *in vitro* effect of BCG. This is consistent with evidence from previous studies in which BCG has been shown to activate monocytes and DCs and stimulate TNF-α production *in vitro* [43], [44]. This observation is critically important for the interpretation of results investigating the immune response to BCG immunisation. Many studies investigating the immune response to BCG or candidate novel TB vaccines also use BCG as an antigen for *in vitro* stimulation. Thus, effects induced by immunisation may not be distinguishable from those resulting from *in vitro* stimulation with BCG. This finding also underlines one of the strengths of the study reported here, namely the availability of results from samples taken both before and after BCG immunisation. The findings also highlight the importance of the choice of *in vitro* stimulatory antigen used to investigate the immune response after BCG immunisation, as responses to BCG and PPD differed for several cytokines.

The absence of significant differences between children and adults in almost all the immunological measures in our study suggests that children in their first year of life and adults have a comparable immune response induced by BCG. Reported differences in protective efficacy between children and adults may therefore be the result of a longer time interval since BCG immunisation in adults, and consequently waning protection. The protective efficacy of BCG differs for different manifestations of TB and is best against severe and disseminated forms that predominantly affect children under the age of two years (up to 73% for TB meningitis and 78% for miliary TB) [1], [2]. An alternative explanation for observed differences in protection between adults and children could therefore be that BCG provides better protective efficacy against the disseminated forms of TB that occur more frequently in infants and young children.

One potential limitation of this study is the multiple statistical tests done to compare the proportions of cytokine-secreting T cells and the concentrations of cytokine and chemokines in supernatants. The possibility of a statistically significant result arising by chance was taken into account by Bonferroni corrections. In addition, the relatively low p-values obtained for many of the markers investigated make this possibility unlikely. Another potential limitation is the relatively small sample size and variable age of the children. However, the assessment of the immune response in the same study at the same time interval after BCG immunisation in both children and adults, and the availability of paired samples (before and after BCG immunisation) are strengths that allow several interesting and important conclusions.

Our study shows that the cell-mediated mycobacterial-specific immune response induced by BCG immunisation in children and adults is comparable. The implication of a shift to a more polyfunctional immune response within the Th1-cytokine-producing CD4 T cell population in children is uncertain as this facet of the mycobacterial-specific immune response has not been assessed as a potential correlate of protection against TB.

## Supporting Information

Figure S1 **Concentrations of cytokines and chemokines in supernatants from children before (grey) and 10 weeks after BCG immunisation.** Whole blood of 13 children was incubated for 7 hours in the presence of the antigens BCG or PPD. Values for BCG- and PPD-stimulated samples were corrected by subtracting the concentration in the unstimulated (NIL) sample. Statistical differences with p-values <0.05 are shown in red. Bars indicate medians.
(TIF)

Figure S2 **Concentrations of cytokines and chemokines in supernatants from adults before (grey) and 10 weeks after BCG immunisation.** Whole blood of 13 adults was incubated for 7 hours in the presence of the antigens BCG or PPD. Concentrations for BCG- and PPD-stimulated samples were corrected by subtracting the concentration in the unstimulated (NIL) sample. Statistical differences with p-values <0.05 are shown in red. Bars indicate medians.
(TIF)
