# Supplementary figures and images for: A Comparative Analysis of Polyfunctional T Cells and Secreted Cytokines Induced by Bacille Calmette-Guérin Immunisation in Children and Adults
Source: PLoS One. 2012 Jul 19;7(7):e37535. doi: 10.1371/journal.pone.0037535 (PMC3400612; doi:10.1371/journal.pone.0037535)

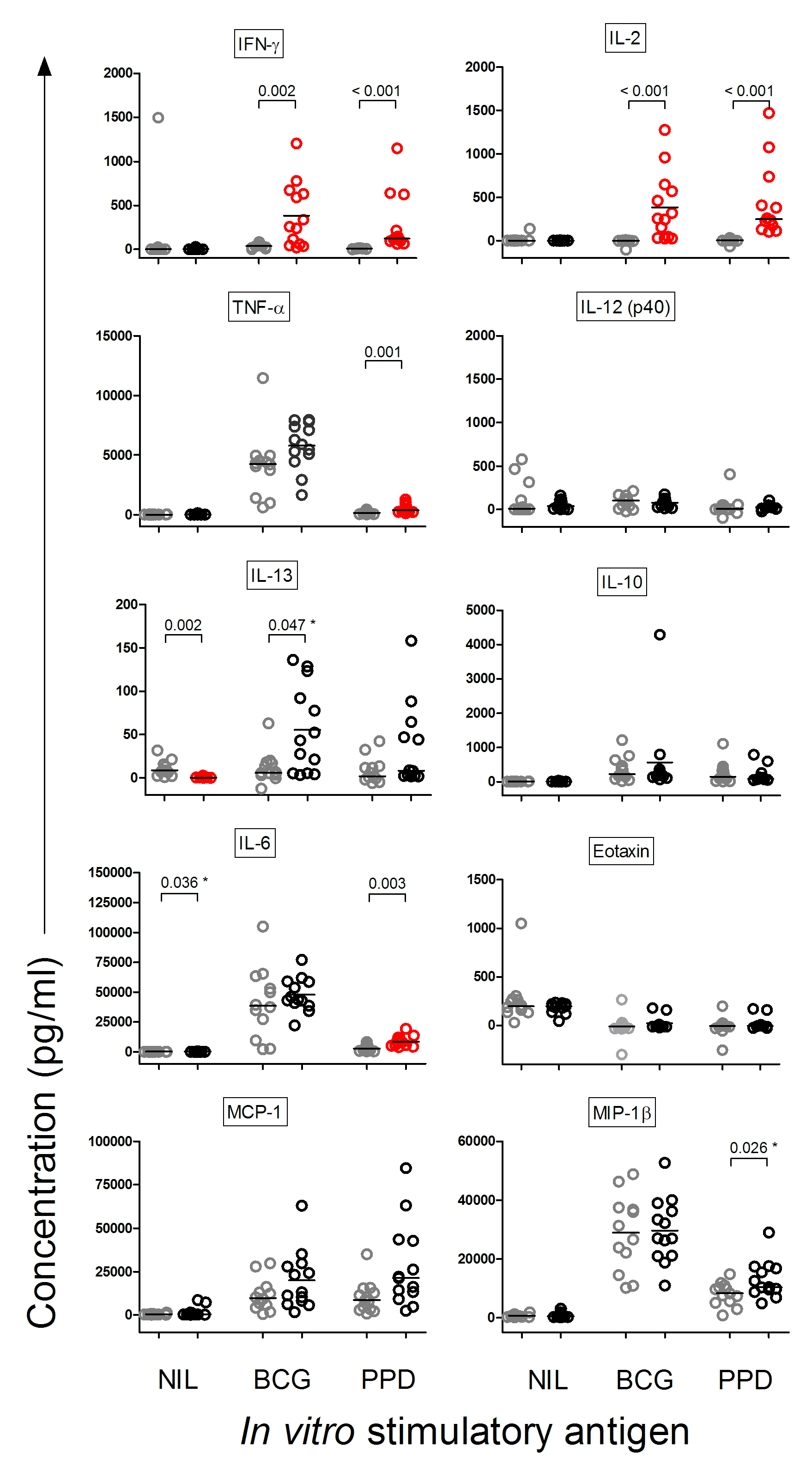

Supplement: Figure S1 — Concentrations of cytokines and chemokines in supernatants from children before (grey) and 10 weeks after BCG immunisation. Whole blood of 13 children was incubated for 7 hours in the presence of the antigens BCG or PPD. Values for BCG- and PPD-stimulated samples were corrected by subtracting the concentration in the unstimulated (NIL) sample. Statistical differences with p-values <0.05 are shown in red. Bars indicate medians. (TIF) [file pone.0037535.s001.tif]

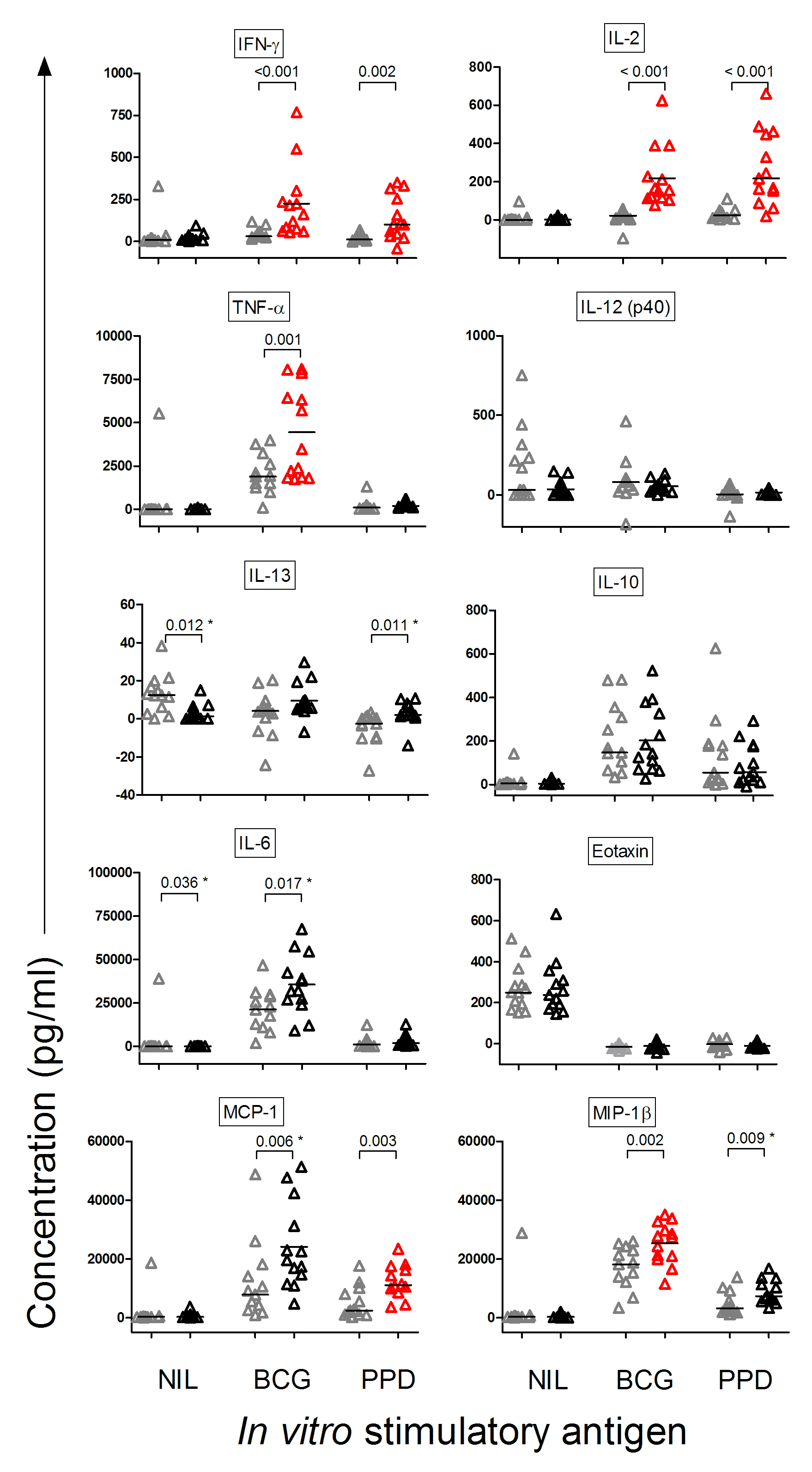

Supplement: Figure S2 — Concentrations of cytokines and chemokines in supernatants from adults before (grey) and 10 weeks after BCG immunisation. Whole blood of 13 adults was incubated for 7 hours in the presence of the antigens BCG or PPD. Concentrations for BCG- and PPD-stimulated samples were corrected by subtracting the concentration in the unstimulated (NIL) sample. Statistical differences with p-values <0.05 are shown in red. Bars indicate medians. (TIF) [file pone.0037535.s002.tif]
